# Evidence for *ACTN3* as a Speed Gene in Isolated Human Muscle Fibers

**DOI:** 10.1371/journal.pone.0150594

**Published:** 2016-03-01

**Authors:** Siacia Broos, Laurent Malisoux, Daniel Theisen, Ruud van Thienen, Monique Ramaekers, Cécile Jamart, Louise Deldicque, Martine A. Thomis, Marc Francaux

**Affiliations:** 1 Exercise Physiology Research Group, Department of Kinesiology, Faculty of Kinesiology and Rehabilitation Sciences, KU Leuven, Heverlee, Belgium; 2 Physical Activity, Sports & Health Research Group, Department of Kinesiology, Faculty of Kinesiology and Rehabilitation Sciences, KU Leuven, Heverlee, Belgium; 3 Institute of Neuroscience, Université catholique de Louvain, Louvain-la-Neuve, Belgium; 4 Sports Medicine Research Laboratory, Luxembourg Institute of Health, Grand-Duchy of Luxembourg, Luxembourg; Victoria University, AUSTRALIA

## Abstract

**Purpose:**

To examine the effect of α-actinin-3 deficiency due to homozygosity for the *ACTN3* 577X-allele on contractile and morphological properties of fast muscle fibers in non-athletic young men.

**Methods:**

A biopsy was taken from the *vastus lateralis* of 4 RR and 4 XX individuals to test for differences in morphologic and contractile properties of single muscle fibers. The cross-sectional area of the fiber and muscle fiber composition was determined using standard immunohistochemistry analyses. Skinned single muscle fibers were subjected to active tests to determine peak normalized force (P_0_), maximal unloading velocity (V_0_) and peak power. A passive stretch test was performed to calculate Young’s Modulus and hysteresis to assess fiber visco-elasticity.

**Results:**

No differences were found in muscle fiber composition. The cross-sectional area of type II_a_ and II_x_ fibers was larger in RR compared to XX individuals (*P*<0.001). P_0_ was similar in both groups over all fiber types. A higher V_0_ was observed in type II_a_ fibers of RR genotypes (*P*<0.001) but not in type I fibers. The visco-elasticity as determined by Young’s Modulus and hysteresis was unaffected by fiber type or genotype.

**Conclusion:**

The greater V_0_ and the larger fast fiber CSA in RR compared to XX genotypes likely contribute to enhanced whole muscle performance during high velocity contractions.

## 1. Introduction

The sarcomeric α-actinins are important structural components of the Z-line where they form the cross-link between the thin actin filaments from adjacent sarcomeres [1]. In addition to their structural function, α-actinins also interact with several structural, signaling and metabolic proteins. In humans, two genes encode for skeletal muscle α-actinins: *ACTN2*, which is expressed in all skeletal muscle fibers and *ACTN3* of which the expression is restricted to type II fibers [2], predominantly involved in powerful explosive contractions.

Worldwide, an estimated 16% of the human population is completely α-actinin-3 protein deficient due to homozygosity for a common stopcodon polymorphism in the *ACTN3* gene called R577X (rs1815739) [3]. This deficiency does not result in a disease phenotype or muscular functional impairment, likely through a compensatory upregulation of the homologous α-actinin-2 protein. However, a number of studies have provided data to indicate that there is a strong association between the *ACTN3* R577X polymorphism and performance in multiple athletic cohorts. The R-allele has been found to be overrepresented in power athletes compared to the average population [4,5]. In non-athletic populations, young RR men perform better on tasks with an explosive strength component compared to XX genotypes, including short sprints, squat jump and fast isokinetic movements [6,7,8], although not all studies confirm these genotype-dependent differences [9,10,11,12].

The mechanisms by which α-actinin-3 alters skeletal muscle function are still under study. The absence of α-actinin-3 in fast (type II) fibers may change the contractile and/or morphological properties of these fibers and consequentially the characteristics of the whole muscle, including maximal force, the force-velocity relationship and muscular fatigability. The *Actn3*^*-/-*^ knockout mouse model has provided evidence for this theory. At the whole muscle level, the KO mice show lower muscle mass, decreased muscle strength, a longer time to exhaustion and an enhanced recovery from fatigue, alterations which are thought to be the result of observed changes at the single fiber level [13,14]. The KO fast muscle fibers have a higher activity of aerobic enzymes, a delayed Ca^2+^ loading of the sarcoplasmic reticulum and a smaller diameter compared to fast fibers of wild mice [13,14].

A previous study in our lab has already determined differences in single muscle fiber characteristics between *ACTN3* R577X genotypes in three individuals with spinal cord injury (SCI). The R-allele carriers showed a higher maximal unloaded velocity and a higher stiffness in their type II_a_/II_x_ fibers compared to the XX genotype [15]. Yet, it has to be noted that immobilized muscles of individuals with SCI undergo marked changes [16], such as notably lower muscle mass and a higher percentage of type II fibers compared to able-bodied individuals. Also, their type II fibers are smaller, but have a greater contraction velocity and force production compared to able-bodied controls [17]. Therefore, the differences between RR and XX genotypes found in individuals with SCI can probably not be extrapolated to able-bodied individuals.

This study therefore aims to analyze skinned single muscle fibers to get more insight into the contractile and morphological properties of α-actinin-3 deficient and α-actinin-3 expressing muscle fibers in non-athletic young males. No differences are expected in type I fibers as α-actinin-3 is only present in type II fibers. Therefore, these fibers can be used as controls to certify equality between groups. Based on the hypothesized function of the α-actinin-3 protein and the properties of type II fibers, it is expected that fast fibers of RR individuals will have a higher maximal force output and a greater (maximal) velocity of shortening compared to XX fast fibers. Also, the diameter of XX fast fibers is hypothesized to be smaller, based on KO mice showing a smaller type II_b_ fiber diameter. The higher stiffness of RR fast fibers in the paraplegic study may also be present in this group of able-bodied young men.

## 2. Methods

### 2.1 Participants

Eight non-athletic young men (age 20.9±0.7 yr) were selected out of a total group of 266 participants based on their characteristics, including stature, weight, physical activity level and maximal isometric knee extension torque at 45° knee flexion (Table 1). Each RR participant was matched with the XX participant that most resembled the results on the previous characteristics. Physical activity level was determined during an interview to determine the amount and type of activities of the participants. Besides sports activities at recreational level, all subjects reported low levels of physical activity as university/high-shool students during daytime and leisure time. Static (45° knee flexion) and concentric (300°/s) torques of the knee-extensor muscles were determined on a self-constructed computerized active isokinetic dynamometer (servomotor SEW Eurodrive CM90, Bruchsol, Germany) as previously described [7]. Cross-sectional area of the quadriceps muscle was estimated based on the circumference of the midthigh corrected for subcutaneous fat at this location using the equation of Housh et al. [18]. Informed consent was obtained from all individual participants included in the study. All participants volunteered and gave written consent to participate in this study, the protocol of which had been approved by the Medical Ethics Committee UZ KU Leuven. All experiments complied with the principles of the Declaration of Helsinki and its subsequent amendments.

**Table 1:** Characteristics of the *ACTN3* 577RR and 577XX genotype groups.

	RR n = 4	XX n = 4	*P-value*	ES	CI
Age, yr	20.1±1.8	21.7±2.2	0.29	-0.71	[-2.14–0.72]
Stature, cm	176.2±4.4	174.5±7.8	0.71	0.24	[-1.15–1.63]
Weight, kg	68.4±8.6	65.7±11.4	0.72	0.23	[-1.16–1.62]
CSA m. quadriceps	62.5±13.3	61.5±1.5	0.89	0.09	[-1.30–1.47]
Sport participation, h/week	4.1±1.4	4.6±2.2	0.72	-0.46	[-1.86–0.95]
Isometric knee torque at 45°, Nm	180±35	182±31	0.95	-0.04	[-1.43–1.34]
Concentric knee torque at 300°/s, Nm	77±23	54±6	0.10	1.18	[-0.32–2.69]

Values are mean±SD. CSA = cross-sectional area; ES = unbiased Effect Size; CI = 95% confidence interval of the ES

### 2.2 Muscle Biopsies

The needle biopsy method with suction was used to obtain a sample from the *vastus lateralis* of the left leg. All participants were asked to retain from intense physical activity three days before the biopsy. A small part of the muscle biopsy was embedded in Tissue-Tek and frozen in nitrogen-cooled isopentane for immunohistochemical analyses. Muscle cryosections were immunohistochemically stained for myosin heavy chain isoforms determination as previously described [7]. The samples used for single fiber testing were immediately placed in cold (0°C) skinning solution and sectioned longitudinally in small bundles of fibers [19]. The bundles were stored in regularly replaced skinning solution at –20°C for at least 5 days before the first experiment. Composition of all solutions can be found in Malisoux et al. (2007).

### 2.3 Single Muscle Fiber Experiments

After skinning, single muscle fibers were evaluated either for peak activated force normalized for fiber cross-sectional area (P_0_), maximal unloaded velocity (V_0_) and force-velocity relationship or passive tension characteristics on a dynamic force set-up. All experiments were performed at 15°C within four weeks after the biopsy was taken. Detailed protocols have been previously described [17,19]. In short, single fiber dimensions including fiber length and cross-sectional area (CSA) were determined on a numeric picture of the fiber after adjustment of sarcomere length to 2.5 μm. Each fiber was visually checked for damage due to cutting or handling of the muscle biopsy resulting in the elimination of damaged fibers prior to testing to exclude an effect on fiber contractile properties. P_0_ was determined as the stable maximal force developed by the fiber while submerged in activating solution (pCa 4.5) corrected for fiber CSA. The unloaded shortening velocity (V_0_) was measured by the slack test; each fiber was maximally activated and then rapidly shortened, such that force fell to baseline and redeveloped after a time lapse, proportional to the step length. Fiber V_0_ was determined as the slope of the fitted line and was expressed in fiber lengths per second (FL/s) to account for differences in the number of sarcomeres in series between different fibers tested. To determine the force-velocity relationship, the fiber was subjected 5–6 times to three successive isotonic load clamps. The data obtained on a single fiber were fitted using an iterative nonlinear curve-fitting procedure (Marquardt-Levenberg algorithm) based on the Hill equation. Fiber power (W/l) was determined from the parameters of the fitted force-velocity curve relationship. Passive tension was tested with a progressive stretch-release protocol while the fiber remained in the pCa 9.0 solution. Young's modulus (kN/m^2^) and hysteresis (kN/m^2^) were calculated to assess visco-elastic properties of the fiber. After completion of the mechanical tests, the fiber segment was dissolved in 25 μl of SDS sample buffer, and stored at –20°C until analyzed for MHC isoform content using SDS-PAGE as previously described [20].

### 2.4 Genotyping

DNA was extracted from EDTA blood samples at UZ Leuven using chemagic Magnetic Separation Module I following the protocol of the manufacturer (PerkinElmer, Baesweiler, Germany). Genotyping of the *ACTN3* R577X polymorphism (rs. 1815739) was performed using a TaqMan SNP genotyping assay (ID C__590093_1, Applied Biosystems). Real-time qPCR was carried out in a 20 μl reaction mixture with 1 μl DNA, 8 μl RNase-free water, 1 μl of 20× TaqMan SNP genotyping assay mix, and 10 μl of the 2× Taqman universal PCR master mix (Applied Biosystems). All reactions were set up manually, and allele calling was done using SDS 1.3 software and visually checked.

### 2.5 Statistical Analyses

Participant characteristics and muscle fiber composition were compared between *ACTN3* 577RR and 577XX individuals using a Student’s t-test. Single fiber measurements were compared per fiber type between the two genotype groups using ANOVA analyses in SAS version 9.2 (SAS Institute, Cary, NC). A proc mixed procedure with a multilevel model was used to account for dependency of multiple fibers within one participant. Unbiased effect sizes and 95% confidence intervals for the genotype differences are reported. Only type I and type II_a_ fibers were numerous enough to allow statistical analyses. Data for hybrid fibers is presented in S1 Table. Scores beyond three standard deviations from the mean were considered to be outliers. These scores are discarded in further analyses. Only one test result obtained from a type II_a_ fiber from an RR participant was excluded with a Young’s Modulus of 213.2 kN/m² (Total group mean = 20.5 kN/m²).

Optimal Design Plus Empirical Evidence version 3.01 (William T. Grant Foundation, NY, NY) was used to estimate the power of the study.

## 3. Results

### 3.1 Participant characteristics

Participant characteristics for the matched *ACTN3* 577RR and 577XX participants are presented in Table 1. No differences were found in anthropometric measurements between both groups. Furthermore, the hours of sport practice per week and the isometric knee torque were similar between both groups.

### 3.2 Muscle fiber composition

A total of 1203 fibers were analyzed with an average count of 150 fibers per participant ranging from 54 to 178 fibers. On average, the relative surface area of type I, type II_a_ and type II_x_ fibers was 53% (25.6–64.5%), 41.5% (23.5–65.6%) and 5.5% (0.5–12.0%) respectively in the total group (Table 2). No genotype-specific differences were found for the relative surface area or the proportion of the three fiber types. The cross-sectional area (CSA) of type II_a_ and type II_x_ fibers was significantly larger in the RR compared with the XX participants. The CSA of type I fibers were similar for both groups (Table 2).

**Table 2:** Type I, type II_a_ and type II_x_ muscle fiber properties of *ACTN3* 577RR and 577XX genotypes.

	RR	XX	*P*-value	ES	CI
*Type I*					
Proportion, %	56±9	53±5	0.79	0.18	[-1.23–1.54]
Relative surface area, %	53±9	53±4	0.96	0.03	[-1.36–1.42]
CSA, μm²	2993±53	2968±51	0.74	0.03	[-0.12–0.17]
P_0_, kN/m²	163.7±5.3	161.3±5.2	0.53	0.02	[-0.43–0.46]
V_0_, FL/s	0.97±0.05	1.12±0.03	0.59	-0.54	[-0.98 –-0.11]
Peak power, W/l	3.03±0.22	2.98±0.11	0.50	0.05	[-0.40–0.49]
Young’s modulus, kN/m²	23.7±1.9	21.2±4.4	0.99	0.14	[-0.39–0.67]
Hysteresis, kN/m²	1.64±0.19	1.55±0.36	0.99	0.06	[-0.61–0.50]
*Type II*_*a*_					
Proportion, %	38±8	42±4	0.68	-0.24	[-1.63–1.15]
Relative surface area, %	40±9	43±3	0.82	-0.12	[-1.51–1.26]
CSA, μm²	4312±129	3207±46	<0.001	0.84	[0.33–1.34]
P_0_, kN/m²	210.1±7.0	184.9±4.8	0.32	0.73	[0.25–1.19]
V_0_, FL/s	4.07±0.18	3.25±0.16	0.001	0.74	[0.28–1.19]
Peak power, W/l	13.5±0.7	10.1±0.6	0.26	0.86	[0.39–1.33]
Young’s modulus, kN/m²	19.3±1.7	21.7±1.7	0.34	-0.32	[-0.92–0.32]
Hysteresis, kN/m²	1.22±0.17	1.21±0.13	0.95	0.02	[-0.60–0.64]
*Type II*_*x*_					
Proportion, %	6±2	5±2	0.68	0.24	[-1.16–1.63]
Relative surface area, %	6±2	5±2	0.61	0.33	[-1.07–1.72]
CSA, μm²	3982±202	2943±107	<0.001	1.21	[1.01–1.42]
P_0_, kN/m²	223.9±16.2	189.4±15.0	0.35	0.80	[-0.69–2.80]
V_0_, FL/s	5.66±0.71	5.09±0.76	0.89	0.33	[-1.00–1.65]
Peak power, W/l	18.8±8.3	9.1±3.1	0.29	1.21	[-0.34–2.75]
Young’s modulus, kN/m²	n.a.	18.0±1.3	n.a.	n.a.	n.a.
Hysteresis, kN/m²	n.a.	1.25±0.22	n.a.	n.a.	n.a.

Values are means±SE; ES = unbiased effect size; CI = 95% confidence interval of ES; n.a. = not applicable

### 3.3 Fiber type-specific P_0_ and V_0_

In total, 183 fibers were actively tested of which 79 type I, 3 type I/II_a_, 80 type II_a_, 12 type II_a_/II_x_ and 9 type II_x_ fibers. Overall, peak force normalized for CSA (P_0_) of type II_a_ fibers was 21% higher than in type I fibers (*P*<0.001). Also, the maximal unloaded velocity (V_0_) was significantly higher in type II_a_ fibers compared to type I fibers (*P*<0.001).

P_0_ and V_0_ in type I fibers were similar in *ACTN3* 577RR and 577XX genotypes (*P* = 0.53; *P =* 0.59). In type II_a_ fibers, V_0_ was significantly higher in the RR compared to XX genotypes (*P* = 0.001). However, P_0_ did not differ between groups (*P* = 0.32)(Table 2).

**Fig 1:**
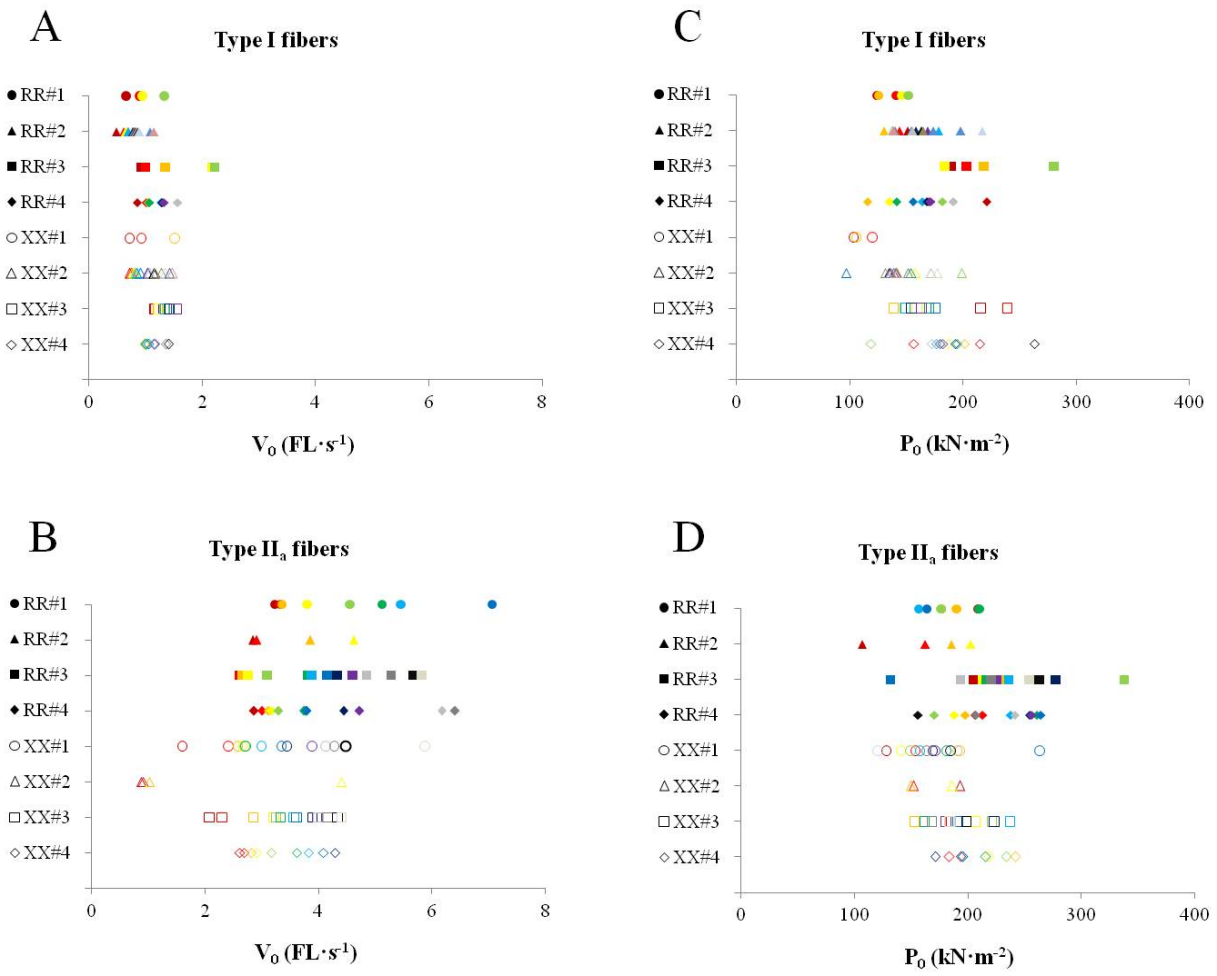
Results for V_0_ (FL/s) of type I (A) and type II_a_ fibers (B), as well as for normalized force (kN/m²) of type I (C) and type II_a_ fibers (D) displayed as per participant. RR = filled markers, XX = blank markers. Matched participants have the same marker shape. Each individual fiber has the same marker color in panel A and C and panel B and D.

Although force was corrected for fiber CSA, there was a high variability in P_0_ within each participant in type I fibers as well as in type II_a_ fibers. Participant RR#3 showed the highest range in P_0_ with scores from 131.7 to 338.2 kN/m² in his type II_a_ fibers. The highest variability in V_0_ was found in type II_a_ fibers of participant RR#1 with values ranging from 3.23 to 7.07 FL/s (Fig 1).

**Fig 2:**
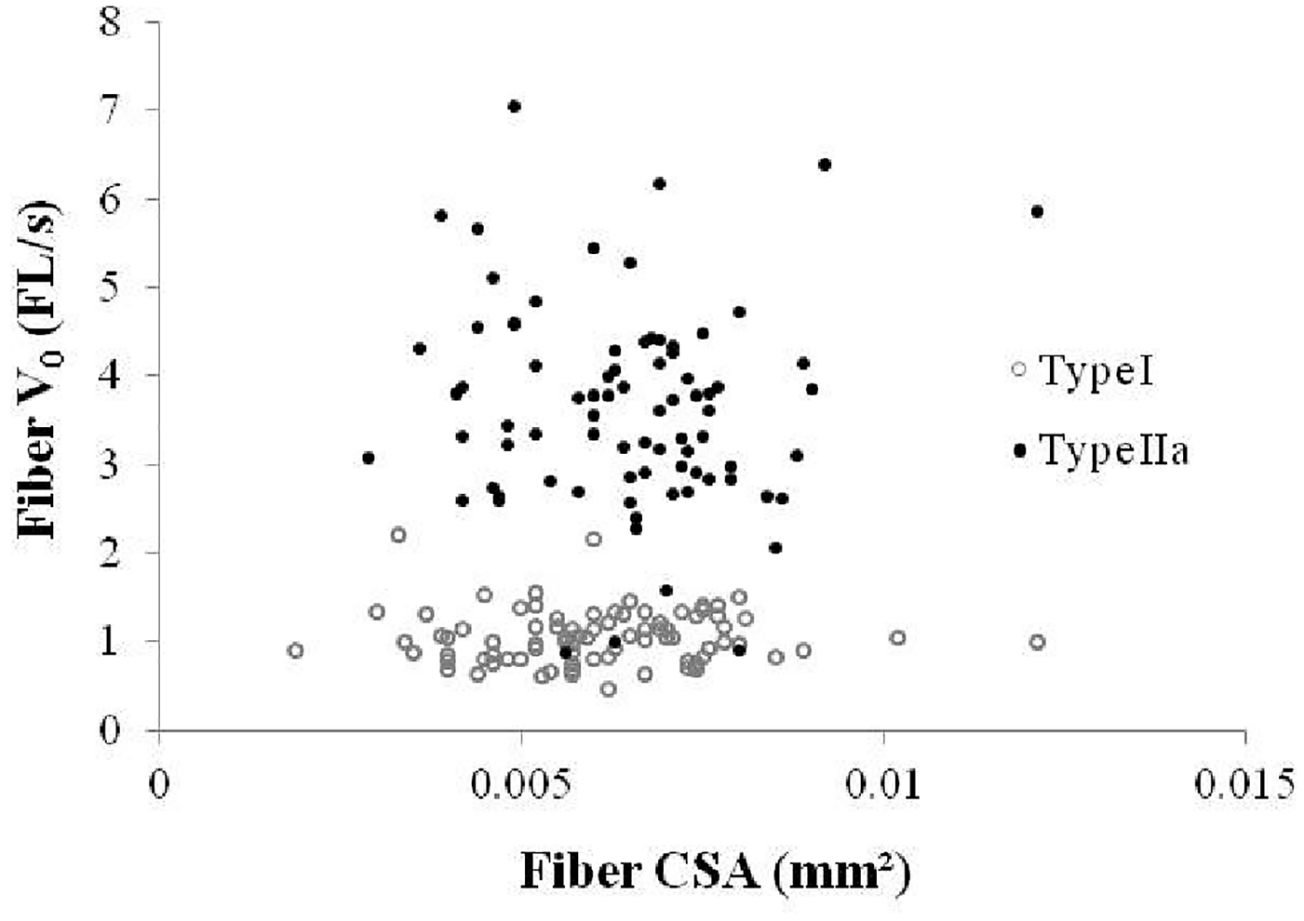
Correlation between V_0_ (FL/s) and fiber CSA of type I (A) and type II_a_ fibers (B).

In addition, no correlation was found between fiber CSA and V0 in type I (r = 0.01) nor in type IIa fibers (r = 0.04) (Fig 2).

### 3.4 Peak power

Overall, peak power was significantly higher in type II_a_ compared to type I fibers (*P*<0.001). There was no difference between *ACTN3* 577RR and 577XX genotypes in peak fiber power in type I (*P* = 0.50) nor in type II_a_ fibers (*P* = 0.26).

### 3.5 Passive tension

Passive tension characteristics of single fibers were assessed on a total of 116 fibers of which 54 type I, 43 type II_a_, 13 type II_a_/II_x_ and 6 type II_x_ fibers. No differences were found between type I and type II_a_ fibers in the total group regarding the visco-elasticity as determined by Young’s Modulus (*P* = 0.53) and hysteresis (*P* = 0.12). When compared between *ACTN3* R577X genotypes, hysteresis and Young’s Modulus were similar in both type I and II_a_ fibers (0.34<*P*<0.99) (Table 2).

## 4. Discussion

This study aimed to explore contractile and morphological properties in single muscle fibers and compare those fiber-type specific characteristics between *ACTN3* 577RR and 577XX homozygotes to explain the influence of α-actinin-3 deficiency on whole muscle force. Overall, the observed values of force, velocity, power and visco-elasticity in skinned muscle fibers were similar to those previously reported [17,21,22]. As expected, force, velocity and power of type II_a_ fibers were higher compared to type I fibers. No genotype-dependent difference was observed in any of the measured variables of type I fibers, which is in accordance with the restricted expression of α-actinin-3 to type II fibers.

The absence of α-actinin-3 in fast muscle fibers has been associated with reduced muscle force, especially during high velocity contractions [7,23,24,25]. Although the concentric knee torque at 300°/s was on average 11% higher (*P*<0.05) in RR versus XX subjects in the total group of 266 participants [23], the 42.5% higher concentric knee torque (300°/s) in the RR genotypes in this study was non-significant due to a high variability in this variable within genotype groups.

Both morphologic and contractile components of the muscle can influence the concentric force production. For instance, whole muscle force is strongly determined by fiber CSA, especially fast fiber CSA as these fibers have a higher maximal force per μm^2^ compared to slow fibers. In this study, type II_a_ and II_x_ fibers of *ACTN3* 577RR genotypes were 14% and 13% larger than 577XX fibers respectively. Despite the increased fast fiber diameter, no differences were found in relative surface area of fast fibers between the two groups, which is in line with Norman et al. (2009)[11], yet in contrast with Vincent et al. (2007)[7]. With similar proportion of slow and fast fibers, larger fast fibers lead to a larger whole muscle CSA. Although the estimated CSA of the quadriceps muscles in this study was similar between genotype groups, several studies using reference methods observed larger CSA or volume in α-actinin-3 expressing muscles of humans and mice [8,13,25,26]. Thus, the larger fast fiber CSA may lead to a greater muscle volume which may contribute to the higher concentric force production in RR genotypes.

If the advantage of α-actinin-3 during strength tests was purely related to muscle volume, a higher isometric force would be expected in RR compared to XX genotypes. Nevertheless, most studies did not find an effect of the *ACTN3* R577X polymorphism on isometric strength of the leg extensors [7,10,11,23,25,27], suggesting that apart from the higher muscle volume, contractile properties of the muscle may be altered by the *ACTN3* R577X polymorphism. The higher concentric torque [7,23] and the similar isometric force between genotype groups indicate an influence of α-actinin-3 deficiency on the preservation of force with increasing velocity rather than on force itself. Indeed, no differences were found in peak force of isolated type I and type II_a_ fibers between genotype groups in this study. In contrast, V_0_ of type II_a_ fibers was higher in *ACTN3* 577RR compared to 577XX genotypes.

Although the velocity of a muscle fiber mainly depends on its MHC isoform, there is a wide variability in V_0_ within a single fiber type [28]. At present, no definitive explanation is available for this variability, although many potential causes have been considered. An effect of myosin light chain (MLC) isoforms composition on single muscle fiber velocity has been observed in rats [29] and humans [30]. Another possibility is the modulation of the functional properties of myosin [31] by myofibrillar proteins such as titin, troponin and Myosin binding protein C. Isoforms of α-actinins have been correlated with different expression levels of troponin [32] and altered binding affinity to titin [33]. Although α-actinin-3 interacts with these proteins, the present data cannot provide evidence that this is the mechanism by which α-actinin-3-deficiency influences V_0_.

Fiber V_0_ is an important determinant of the maximal shortening velocity of the muscle, a parameter of the force velocity curve. The force-velocity relationship (FVR) is the most critical muscle contractile property in the limitation of maximal human sprinting speed [34]. A "velocity-oriented" force-velocity profile is one of the main mechanical determinants of 100-m sprint performance [35]. In line, Mero et al. (1986) showed evidence for a higher importance of variables associated with velocity rather than force production capability during sprint [36]. Thus, the higher fast fiber V_0_ in RR genotypes may improve sprinting ability, explaining why the association of the RR genotype with athletic performance is strongest in sprinters [37,38,39].

No difference was found in the visco-elasticity of the fibers as determined by the variables Young’s Modulus and hysteresis. This is in contrast with the higher stiffness of type II_a_/II_x_ fibers in *ACTN3* 577RR compared to 577XX genotypes in a case series study involving paraplegic individuals [15]. The reason why the higher fast fiber stiffness is present in the paraplegic RR muscle is uncertain, but may be related to the changes in muscle fiber properties during longtime immobilization of the muscle.

Although the statistical analyses were performed on a total of 299 fibers, only eight individuals participated in this study. Therefore, the effect size (ES) for the overall comparisons between XX and RR subjects was reported for each of the measured variables in addition to the p-value. ES is a simple way to show the actual difference, which is independent of the sample size [40]. In this way the meaningfulness of the differences in fiber contractile properties between *ACTN3* 577 RR and XX can be interpreted by the reader (unaffected by the sample size issue). As expected, effect sizes were small for most fiber contractile properties in type I fibers, ranging from 0.02 to 0.29, except for V_0_, which showed a medium ES of -0.54. In contrast, P_0_ and peak power of type II_a_ and II_x_ fibers showed large effect sizes (0.73–1.21), although null hypothesis significance testing using the multilevel approach did not reach significance.

To be thorough, the program Optimal Design Plus Empirical Evidence was used to estimate the post-hoc power of the study. Based on the number of fibers, subjects, means, within-subject and between-subject variability for CSA of type II_a_ fibers, this study reaches a power of 0.49.

Comparisons were performed within a multi-level model approach to account for the dependency of fibers within each participant. In addition, it cannot be ruled out that structural differences exist in thin actin filaments between *ACTN3* R577X genotypes, which could have influenced our results. A decrease in actin filament length has been observed after longtime disuse of the muscle [41]. As in all studies on isolated fibers, we assumed 2.5μm to be the optimal sarcomere length.

In conclusion, this study shows that α-actinin-3 deficiency decreases the contraction velocity of isolated type II_a_ muscle fibers. The increased cross-sectional area of type II_a_ and II_x_ fibers may explain the increased muscle volume in RR genotypes. Thus, our results suggest that, rather than fiber force, combined effects of morphological and contractile properties of individual fast muscle fibers attribute to the enhanced performance observed in RR genotypes during explosive contractions.

## Supporting Information

S1 Table Properties of single muscle fibers with mixed MHC-isoforms in participants with RR and XX genotypes. Values are means±SE. n.a. = not applicable.
(DOCX)
