# Supplementary material for: Evidence for ACTN3 as a Speed Gene in Isolated Human Muscle Fibers
Source: PLoS One. 2016 Mar 1;11(3):e0150594. doi: 10.1371/journal.pone.0150594 (PMC4773019; doi:10.1371/journal.pone.0150594)
Supplement: S1 Table — Values are means±SE. n.a. = not applicable. (DOCX) [file pone.0150594.s001.docx]

| Supplemental Table 1. Properties of single muscle fibers with mixed MHC-isoforms in participants with RR and XX genotypes | | | | |
| --- | --- | --- | --- | --- |
|  | RR | XX |  | *P*-value |
| Type I/II_a_ |  |  |  |  |
| P_0_, kN/m² | 152.5±16.3 | n.a. |  | n.a. |
| V_0_, FL/s | 2.05±0.51 | n.a. |  | n.a. |
| V_max_, FL/s | 2.77±1.67 | n.a. |  | n.a. |
| Young’s modulus, kN/m² | n.a. | n.a. |  | n.a. |
| Hysteresis, kN/m² | n.a. | n.a. |  | n.a. |
|  |  |  |  |  |
| Type II_a_/II_x_ |  |  |  |  |
| P_0_, kN/m² | 187.7±10.9 | 187.7±13.6 |  | 0.95 |
| V_0_, FL/s | 4.10±0.49 | 3.91±0.41 |  | 0.77 |
| V_max_, FL/s | 1.71±0.81 | 1.50±0.20 |  | 0.70 |
| Young’s modulus, kN/m² | 17.9±2.3 | 18.2±3.8 |  | 0.95 |
| Hysteresis, kN/m² | 0.84±0.27 | 1.35±0.48 |  | 0.27 |
| Values are means±SE. n.a. = not appilcable | | | | |
